# The Effect of TiO_2_ Doped Photocatalytic Nano-Additives on the Hydration and Microstructure of Portland and High Alumina Cements

**DOI:** 10.3390/nano7100329

**Published:** 2017-10-14

**Authors:** María Pérez-Nicolás, Íñigo Navarro-Blasco, José M. Fernández, José Ignacio Alvarez

**Affiliations:** MIMED Research Group, Departamento de Química, Facultad de Ciencias, Universidad de Navarra, Irunlarrea, 1, 31008 Pamplona, Spain; mperez.52@alumni.unav.es (M.P.-N.); inavarro@unav.es (Í.N.-B.); jmfdez@unav.es (J.M.F.)

**Keywords:** cement, alumina cement, nano-additives, doped TiO_2_, Scanning Electron Microscopy (SEM), calorimetry, hydration

## Abstract

Mortars with two different binders (Portland cement (PC) and high alumina cement (HAC)) were modified upon the bulk incorporation of nano-structured photocatalytic additives (bare TiO_2_, and TiO_2_ doped with either iron (Fe-TiO_2_) or vanadium (V-TiO_2_)). Plastic and hardened state properties of these mortars were assessed in order to study the influence of these nano-additives. Water demand was increased, slightly by bare TiO_2_ and Fe-TiO_2_, and strongly by V-TiO_2_, in agreement with the reduction of the particle size and the tendency to agglomerate. Isothermal calorimetry showed that hydration of the cementitious matrices was accelerated due to additional nucleation sites offered by the nano-additives. TiO_2_ and doped TiO_2_ did not show pozzolanic reactivity in the binding systems. Changes in the pore size distribution, mainly the filler effect of the nano-additives, accounted for the increase in compressive strengths measured for HAC mortars. A complex microstructure was seen in calcium aluminate cement mortars, strongly dependent on the curing conditions. Fe-TiO_2_ was found to be homogeneously distributed whereas the tendency of V-TiO_2_ to agglomerate was evidenced by elemental distribution maps. Water absorption capacity was not affected by the nano-additive incorporation in HAC mortars, which is a favourable feature for the application of these mortars.

## 1. Introduction

Photocatalytic additives can be added to cementitious materials to pursue the enhancement of their activity and their application in new areas. The efficiency of these photocatalytic cements has been tested in different previous works, showing depolluting activity of the atmosphere and self-cleaning properties [1,2,3,4]. TiO_2_, titania, appears as the most widely active compound used in these cement-based materials [5,6,7]. Under ultraviolet (UV) irradiation, titania has shown an outstandingly good photocatalytic efficiency. However, in order to broaden the sensitivity of the titania towards visible light illumination, the use of doped additives is being explored [8,9,10]. These additives show capacity to absorb visible light photons, allowing the application of photocatalytic materials in areas with low, if any, incidence of UV photons. Doping with transition metals (Fe, Cr, V, etc.) is one of the most studied alternatives [11,12,13].

A previous work has demonstrated the photocatalytic efficiency of two doped nano-additives, Fe-TiO_2_ and V-TiO_2_, when immobilized by physical entrapment onto different cement systems to remove atmospheric NO*_x_* [14]. The choice of these two transition metals, Fe and V, as dopants was based on (i) their well-known efficiency in reducing the band-gap of the TiO_2_ lattice; and (ii) the reliable knowledge of their synthetic route as nanoparticles by flame spray pyrolysis (FSP). In that previous work the use of different binders such as Portland cement and calcium aluminate cements was envisaged in order to apply photocatalytic materials in many different places and to take advantage of different positive synergistic effects that could arise as a consequence of the specific chemical, mineralogical and porous structure of these binders [1,7,15].

However, for their practical application, two issues should be addressed. On the one hand, the nano-additives have to be well spread out in the bulk mortar. In this sense, different strategies to guarantee the scattering of the nano-additives have been discussed, such as the ultra-sonication of the suspensions of the nano-particles and the use of dispersing agents (superplasticizers) [16,17,18,19].

On the other hand, the effect of the incorporation of the nano-structured photocatalytic additives onto the plastic and hardened performance of the mortars has to be carefully assessed. Changes in the properties of the mortars can have a strong influence on the feasibility of the photocatalyst incorporation, such as modifications in the hydration times or in the mechanical strengths. Literature has shown that the incorporation of nano-structured additives within binding mortars modifies the mixing water requirements, thus impacting the mechanical properties [20,21,22,23]. Although bare titania has been described as a non-pozzolanic additive, the presence of TiO_2_ with small particle size has led to an acceleration of the hydration in Portland cement (mortars by increasing the number of nucleation sites [24]. The incorporation of nano-additives with chemical and structural modifications of bare TiO_2_ by doping leads to changes in the properties of the mortars which have not been tackled yet in the literature and that is precisely the main goal of the current work.

This paper focuses on the effect of the incorporation of three different photocatalytic additives as nanoparticles, bare TiO_2_ (as control group) and two doped TiO_2_ with transition metals, Fe-TiO_2_ and V-TiO_2_, onto mortars of two different binders: Portland cement (PC) and high alumina cement (HAC). The current research evaluates the changes of the modified mortars in plastic as well as in hardened states, including a correlation with a detailed examination of the microstructure by pore size distribution measurements and scanning electron microscopy (SEM). All these parameters will be monitored in order to assess the possibilities of the real application of these modified cementing matrices, by taking into account variables such as hydration time, compressive strength, porosity, microstructure and water sorption behavior of the resulting materials.

## 2. Results and Discussion

### 2.1. Influence on the Water Demand

Mineralogical compositions of the binders, obtained by X-ray diffraction (XRD), together with the chemical composition provided in the specification sheets of the manufacturer, are summarized in Table 1. For the nano-additives, anatase and rutile appeared as the unique crystalline compounds in all samples (Table 2). The experimental specific surface values obtained for bare titania and doped additives are summarized in Table 2, as well as particle size of the nano-particles, determined according to the Scherrer equation from the XRD results [25].

Nano-photocatalysts incorporation into mortars directly impacted on the water demand that was needed to keep a similar flowability in the fresh pastes, as given by a fixed spread of 175 ± 10 mm. Fresh mixtures of PC doped with titania and iron nano-additives presented a slight increase in the demand of mixing water, as can be observed from the results collected in Table 3.

**Figure 1:**
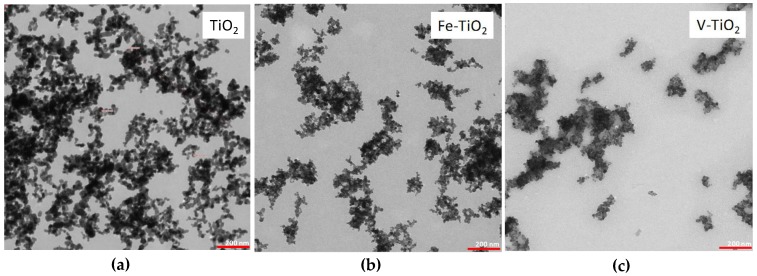
TEM micrographs of the nano-structured additives (×16,000): (**a**) TiO_2_; (**b**) Fe-TiO_2_; (**c**) V-TiO_2_.

This fact can be ascribed to the increase in the adsorption of water as a consequence of the small particle size of these nano-structured additives (see the TEM (transmission electron microscopy) examination of the additives in Figure 1). Low dosages exhibited attenuated changes due also to the filler effect: nano-particles can be distributed among the wide spaces of the pastes, reducing the filling water, with a subsequent lubricating role that could partially counteract the unavailability of the adsorbed water [26].

The use of V-TiO_2_ additive caused an increase in the mixing water requirements, particularly for PC mortars. This finding for the V-TiO_2_ additive can be understood by taking into account its higher specific surface area, owing to its lower particle size (Table 2). The interaction between these fine particles led to the formation of strong van der Waals forces, thus reducing the fluidity of the plastic paste [26].

**Figure 2:**
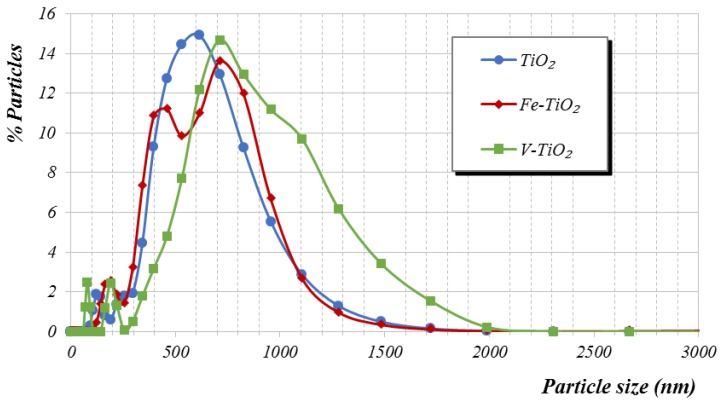
Particle size distribution obtained by light dispersion of the nano-structured photocatalytic additives in an alkaline solution (synthetic pore cement solution, pH 12.5 and 1 wt. % of CaCl_2_).

Measurements of the particle size distribution of the nano-structured additives carried out in pore solution simulating cement media (pH 12.5 and 1% CaCl_2_) confirmed this assumption (Figure 2), evidencing the tendency of V-TiO_2_ to form large agglomerates. V-TiO_2_ has the largest population of agglomerates bigger than 1000 nm in diameter and its main population diameter appeared at 715 nm (while a 615 nm main population diameter was measured for both Fe-TiO_2_ and bare TiO_2_). Zeta potential experimental measurements of the aforementioned simulated cement media were −9, −7 and −5 mV for bare TiO_2_, Fe-TiO_2_ and V-TiO_2_, respectively, which fall within the instability region for all of them. In particular, zeta potential values of V-TiO_2_, with the lowest surface charge, confirmed the tendency of this additive to agglomerate.

These experimental results showed that the incorporation of the bare TiO_2_ and Fe-TiO_2_ nanometrically structured photocatalytic additives under our study slightly modified the water demand of mortars, whereas V-TiO_2_ increased the water demand in agreement with previous works [21,23]. The main conclusion is that the variation of water demand does not follow a linear relationship. This is understandable since the study includes several interlinked factors that hinder the assessment of an isolated factor.

**Figure 3:**
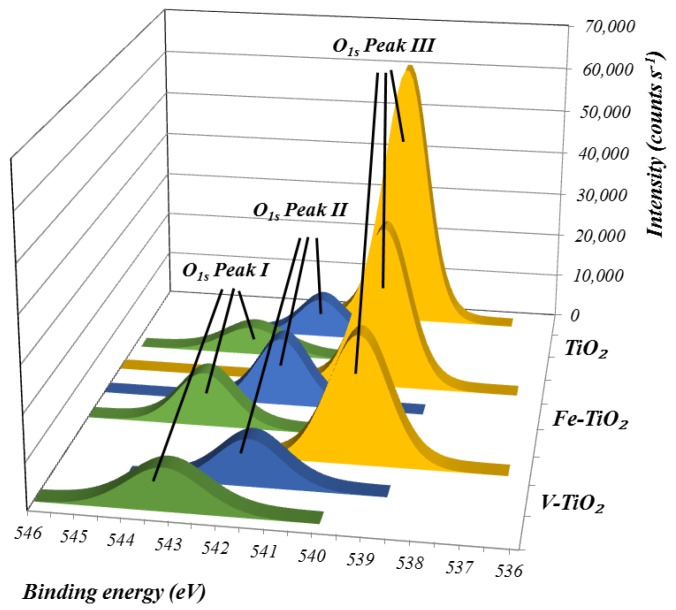
XPS spectra of the O region, showing the three deconvolution peaks of the scans of the assayed photocatalysts. In blue, peak II is related to the adsorption of –OH groups.

Previous X-ray photoelectron spectroscopy (XPS) measurements [14] confirmed that, in line with the precursors used in the synthesis, that the vanadium-bearing additive was a heterostructure between TiO_2_ and VO*_x_* (vanadyl centers) whereas in the case of the Fe-doped additive Fe^3+^ was found to substitute Ti^4+^ within the TiO_2_ lattice. According to the second peak of the deconvolution of the O region (Figure 3), the amount of hydroxyl groups and molecular water adsorption [27] on the surface of the photocatalysts was Fe-TiO_2_ > V-TiO_2_ > bare TiO_2_.

This would imply that Fe-TiO_2_ should be the additive with the highest water retention ability, which is not the case according to the values in Table 3. This apparent contradiction justifies that other concomitant factors such as additive dosage, fineness of the cement particles, particle size distribution of the cement, zeta potential, tendency of the nano-additives to agglomerate, and even the chemical composition of the cement and reactivity play a simultaneous relevant role. To summarize, the overall contribution of these factors showed that PC mortars require the highest percentage of mixing water upon addition of nano-additives when compared with HAC mortars.

### 2.2. Effect on the Hydration Rate and Setting Time

The assessment of the hydration time is necessary in order to prove the feasibility of the bulk incorporation of the additives in these cementing matrices: should the additives induce significant changes in the setting times, their use would be inadvisable.

**Figure 4:**
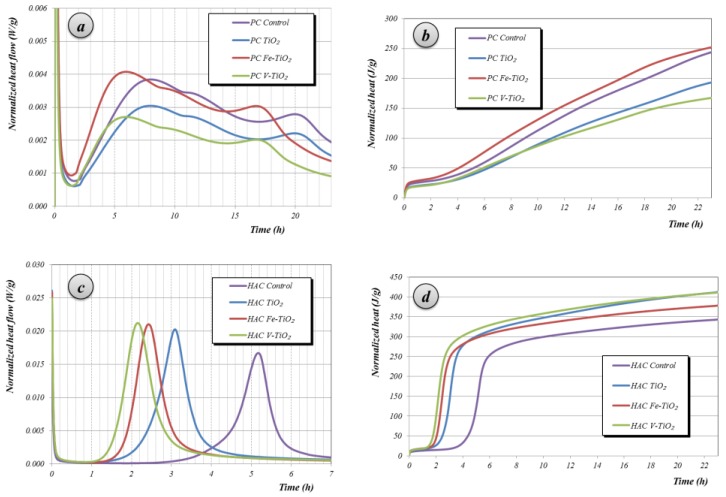
Isothermal calorimetry curves: (**a**,**c**) normalized heat flow and (**b**,**d**) total heat release of PC and HAC pastes with 2.5 wt. % of additives as compared with control (additive-free) sample.

Isothermal calorimetry studies measured the effect of the incorporation of the additives onto the hydration rate of the cementitious phases. These experiments were carried out for pastes, i.e., just binders and water and additives, without aggregate. Accordingly the mixing water amounts differed from those used for mortars and were fixed for each type of cement for comparative purposes. Only the dosage of additive 2.5% by weight of cement is shown (Figure 4) for the sake of clarity and shortness (the additives added in other dosages showed a similar trend).

#### 2.2.1. PC Samples

The experimental curves of PC samples (Figure 4a) showed the strongest heat evolution, which is related to the rapid dissolution of the compounds during the initial minutes after adding the mixing water (highly soluble alkali sulfates and aluminates), early reaction of C_3_A and sulfates to form ettringite as will be described below, and the hydration of free lime [28]. Curves in Figure 4a then showed a strong decrease, ascribed to the first deceleration step, or induction period, leading to a minimum in the heat flow curve. Cement paste remains plastic and workable. The formation of a protective layer on the C_3_S particles has been claimed to be a key factor in this dormant stage [29]. The third stage resulted in a maximum of heat release, related to the hydration of the alite, responsible for the early strength development. During this acceleration period a rapid crystallization of CH (portlandite) and C-S-H (calcium silicate hydrates), corresponding to the main hydration of the PC, took place. Although the linkage of the heat evolution due to the chemical reactions (calorimetric curves) with the setting time (physical formation of the hardened structure) is not linear, the initial setting time corresponds to the time in which the rate of the reaction becomes intense whereas the final setting time would take place before the system reaches the maximum [30,31].

The time at which the maximum of the peak height appeared was affected by the presence of all three additives tested. The occurrence of these peaks was in all instances accelerated by the presence of the photocatalysts, in agreement with the behavior of nano-structured anatase in cement pastes [24]. The peaks appeared at 7.82, 7.25, 6.26 and 5.67 h for plain PC, bare TiO_2_, Fe-TiO_2_ and V-TiO_2_ samples, respectively. The photocatalysts gave rise to an acceleration of the PC hydration of around 34, 93 and 129 min, for bare TiO_2_, Fe- and V-doped TiO_2_, respectively. The incorporation of nanostructured additives promote additional nucleation sites, which increase the speed of the hydration processes, resulting in an acceleration of the cement hydration. In the case of the doped titania, the growth of nucleation sites seems to be more marked, as proved by the stronger acceleration as compared with bare titania. This fact is ascribed to the higher specific surface area (as measured by the Brunauer Emmett Teller (BET) method, Table 2) of the doped additives (Fe-TiO_2_: 101 m^2^ g^−1^; V-TiO_2_: 113 m^2^ g^−1^) as compared with bare TiO_2_ (50 m^2^ g^−1^), confirming the significance of the nucleation sites in the acceleration of the PC hydration, as experimentally proved by the times obtained from the calorimetric curves. This nucleation is thought to take place in a spatially non-random way, only on grain boundaries, according to the Boundary Nucleation model (BN), rather than on randomly distributed locations [24].

Nano-structured titania has been described as a non-pozzolanic additive [32]. The results of the current experimental work with doped nano-structured titania showed that the doped TiO_2_ presented no pozzolanic activity either. The influence of the particle size promoting nucleation sites has been proven as the main factor affecting the time of hydration, since all the cementitious matrices modified with the tested nano-additives showed an acceleration of the heat evolution peaks. However, the chemical modification caused by doping of the additives has also been found to modify the total heat release. The influence of this factor was also confirmed by quantitative TG-DTA (thermogravimetry-differential thermal analysis) results. For example, for PC mortars, the weight losses of water bound to hydraulic components (considering the weight losses between 40 and 200 °C and the loss between 380 and 460 °C that was ascribed to the Ca(OH)_2_ dehydroxilation) [33] were 3.43%, 3.05%, 4.29% and 1.87% for control, bare TiO_2_, Fe-TiO_2_ and V-TiO_2_ samples, respectively.

Results were in good agreement with the total heat release. In spite of the acceleration effect of bare TiO_2_ and V-TiO_2_, their incorporation reduced the amount of hydrates formed during the cement hydration.

#### 2.2.2. HAC Samples

Isothermal calorimetry curves for HAC pastes evidenced a strong influence of the additives (Figure 4c). After the initial wetting of the sample, the main heat evolution was related to the massive precipitation of calcium aluminate hydrates that, in plain HAC paste, took place at 5.16 h after the addition of mixing water. In accordance with results observed for PC pastes, the presence of the photocatalytic additives, providers of nucleation sites, dramatically shortened the induction time (dormant period), and the peaks appeared earlier: 3.13 h for bare TiO_2_, 2.41 h for Fe-TiO_2_ and 2.14 h for V-TiO_2_. The effect of the particle size showed the same trend depicted in PC pastes: the higher the surface area of the additive, the stronger the shortening of the time for the heat release. The semi-quantitative estimation of the XRD phase assemblage of the calcium aluminate phases for these mortars under the two curing conditions is collected in Table 4. The presence of the additives caused a reduction of the anhydrous calcium aluminates, which was proved by the decrease in the intensity of CA and C_12_A_7_ (curing 1). At the same time, the intensities of the diffraction peaks of the hydrated compounds increased, as can be observed for samples subjected to curing 2, with higher amounts of stable calcium aluminate hydrates, such as C_3_AH_6_ (hydrogarnet) and AH_3_ (gibbsite).

### 2.3. Compressive Strengths, Pore Structure and SEM Examination

Compressive strengths were studied to ensure the practical application of these cementing matrices and correlated to the assessment of the microstructure of these mortars by means of the analysis of the pore structure and textural aspect by SEM observations.

#### 2.3.1. PC Mortars

##### Compressive strengths and pore size distribution

**Figure 5:**
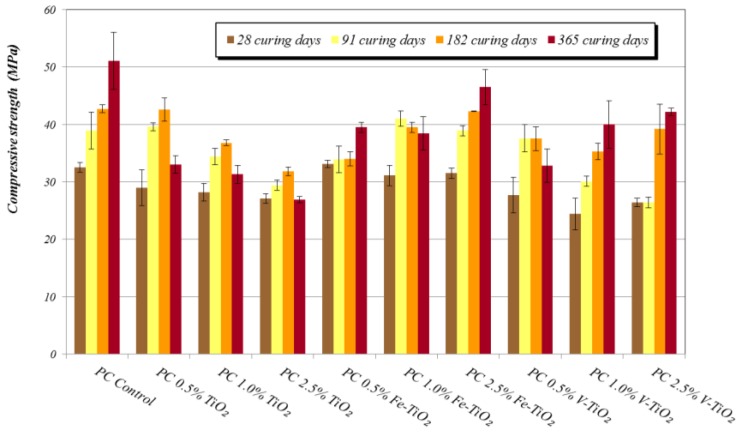
Compressive strengths at different curing times of PC mortars with nano-structured photocatalysts.

Generally, additive-bearing PC mortars exhibited lower compressive strengths than those of plain mortars (Figure 5). Variations in these compressive strength results can be ascribed, for a specific binder, to the influence of several factors, such as: (i) changes in the pore size distribution motivated by the presence of a nanostructured material, which could result in a filler effect (low dosages) or a poor compactness (high dosages), with beneficial or detrimental consequences, respectively; (ii) interferences in the cementitious phases hydration arising from the hydrophilicity of the photocatalytic additives that could withdraw water hindering the complete hydration steps (in this sense, previous comments on the XPS results should be considered); (iii) differences in the amount of mixing water for mortars with each one of the additives.

Contrary to the expected improvement in the compressive strength upon addition of low percentages of nano-additives due to the filler effect, in samples tested after 28 curing days, the increase in mixing water was seen to be the key factor justifying the decrease in the compressive strength values of PC mortars. Among the tested additives, samples with Fe-TiO_2_ yielded the highest compressive strength results depending on the added percentage. V-TiO_2_, with the largest mixing water amount, showed the lowest compressive strengths (28 curing days). V-TiO_2_ samples and Fe-TiO_2_ showed a dosage-dependent pattern: the higher the amount of additive, the higher the compressive strengths. This fact is consistent with the aforementioned expected filler effect owing to the small particle size of these additives, as further discussed below.

**Figure 6:**
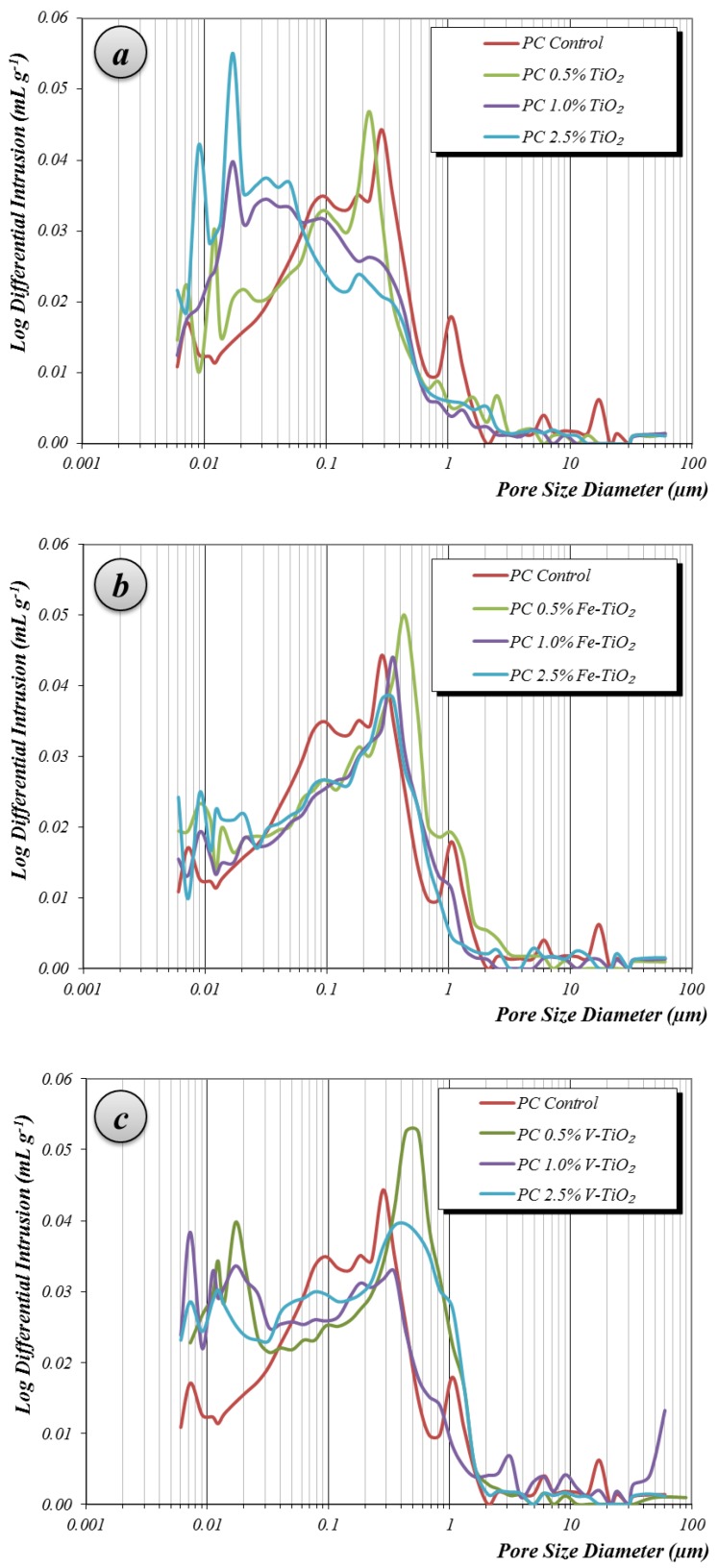
Pore size distribution results of PC samples after 28 curing days for: (**a**) TiO_2_; (**b**) Fe-TiO_2_; (**c**) V-TiO_2_.

Conversely, bare TiO_2_ samples gave rise to a reverse pattern, showing the lowest strengths for the samples with the highest TiO_2_ proportions. This adverse effect can be ascribed to the poor compactness caused by the larger particle size of this additive. Samples at medium-term of curing (28 days) showed a pore size distribution depicting an irregular pattern, but with the main population of pores within the range of large capillaries (pore diameters between 10 and 0.05 μm) (Figure 6a–c) [34]. These pores are responsible for the transport phenomena in the monolithic specimens.

Another significant pore population was found around 0.05 and 0.01 μm, the pore range of the medium capillaries. These pores arise as a consequence of the incorporation of the mineral additives and have a strong effect on the permeability [34]. In the tested samples, it can be observed how the presence of the additives increased the pore population in this range. Increasing amounts of the additives (from 0.5 wt. % to 2.5 wt. %) yielded a higher amount of pores in this range. Among the additives, bare TiO_2_ caused the highest increase in these medium capillaries. At the same time, a parallel reduction in large capillary pores (mainly between 0.05 and 1 μm) can be observed. These changes in the pore size distribution can be explained taking into account the filler effect of the additives (nanometrically structured, but with a strong tendency to agglomerate), increasing the population of the small pores at the expense of the larger ones. The additive with the highest particle size and lowest specific surface area, bare TiO_2_, caused the sharpest changes in the pore size distribution with detrimental consequences to the compressive strengths (poor compactness in the pore range between 0.01 and 0.1 μm).

##### SEM examination

**Figure 7:**
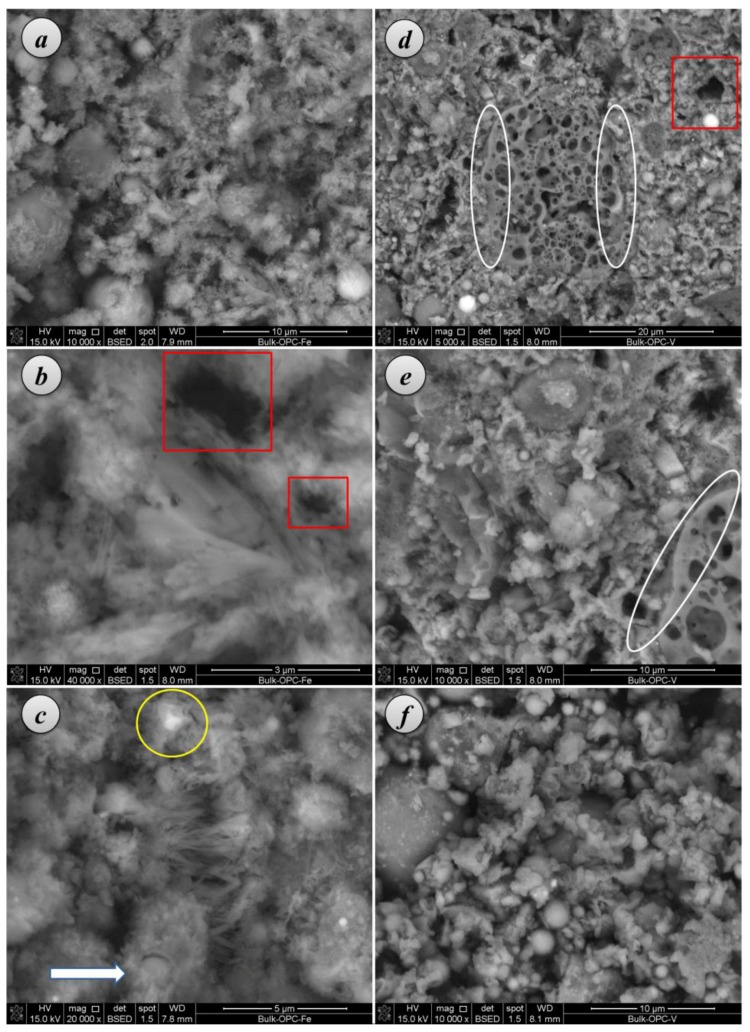
SEM micrographs of PC samples with: 2.5 wt. % of Fe-TiO_2_ (**a**–**c**) and 2.5 wt. % of V-TiO_2_ (**d**–**f**), after 28 curing days.

Microstructural assessment of these mortars was carried out by SEM-EDS (Scanning Electron Microscopy-Energy Dispersive X-Ray Spectroscopy). Micrographs showed that PC mortars with either Fe-TiO_2_ or V-TiO_2_ presented a well interlocked structure, with particles of aggregate embedded in the binding matrix (Figure 7a–f). The ITZ (interfacial transition zone) between binder and aggregate was of much reduced porosity (see oval outlined areas in Figure 7d,e as an example, with large aggregate particles fully embedded in the binding matrix with negligible porosity in the ITZ). The textural aspect was not uniform due to the different forms of hydrated phases of the PC as well as to the presence of the photocatalysts.

**Figure 8:**
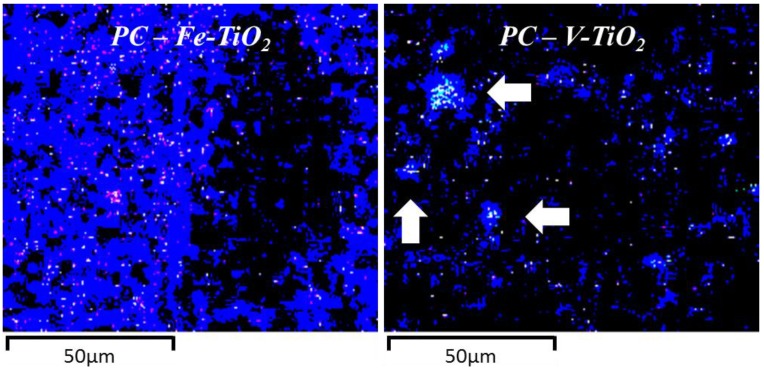
Comparative Ti distribution (SEM-EDS) of PC mortars (after 28 curing days) with Fe-TiO_2_ and V-TiO_2_ nanostructured additives (included in 2.5 wt. %). For the purpose of clarity, due to the low ratio of Ti in the mortars, images were equally adjusted (brightness and contrast). Areas showing an accumulation of Ti in the V-TiO_2_ samples are indicated with arrows.

A detailed examination revealed that a fibrous character was predominant for C-S-H phases [35]. These C-S-H small fibers were also seen to form the external layer of some hollow-shell structures, Hadley grains (1–2 micrometers of diameter), which have been identified as a hydration product of PC and C_3_S pastes [36,37]. Some empty hollow shells can be identified with a slight growth of fibers protruding towards the inner part (see outlined red square areas in Figure 7b,d). In other cases, the inner part of these hollow shells shows unreacted C_3_S that remains un-hydrated (brighter parts in the yellow circled area in Figure 7c). Other Hadley grains were seen to be partially ruptured (see the grain marked with an arrow in Figure 7c). Some fibrils were also identified in the outer part of the C-S-H paste (see the central parts of the Figure 7b,c). No large CH deposits were found in these PC mortars, in good agreement with the small amount ascribed to this phase in TG analysis (dehydroxylation weight loss at *ca*. 480 °C, which was always lower than 0.4%) and in accordance with previous works [35]. Micrographs also show how the C-S-H fibers radiating out in the open space are responsible for the predominant capillary pore structure (see mainly Figure 7a–c), including pores up to 1–2 μm. Nevertheless, large pores are not easily observable as a consequence of the filling effect of the additive, in agreement with the pore size distributions results. High magnification images (Figure 7b,c) show the prevailing presence of medium capillary pores. EDS analysis (elemental mapping) showed a regular distribution of Fe-TiO_2_, whereas V-TiO_2_ confirmed its strong tendency to agglomerate (Figure 8).

#### 2.3.2. HAC Mortars

##### Compressive strengths and pore size distribution

**Figure 9:**
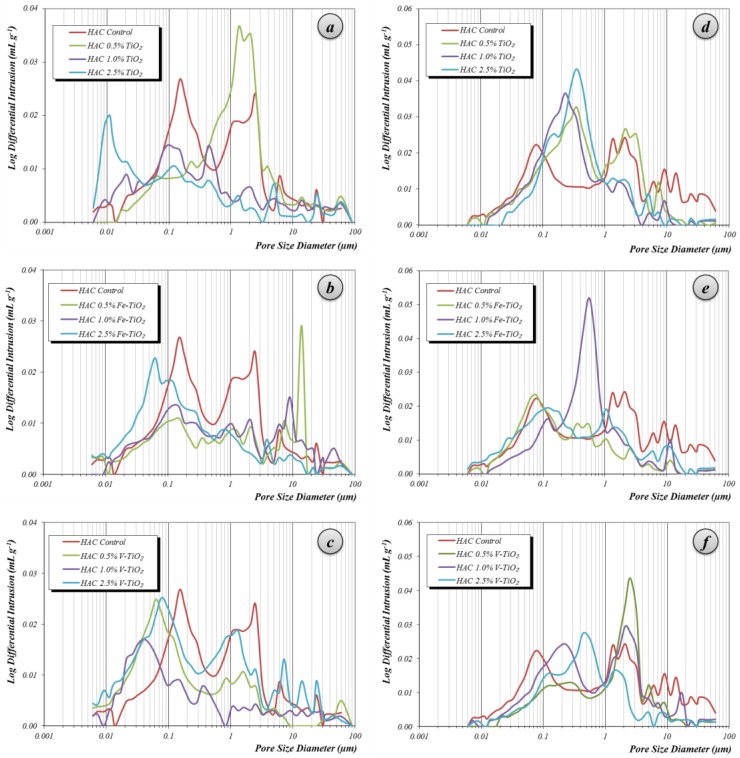
Pore size distribution of HAC mortars with photocatalysts after: (**a**–**c**) 28 curing days at curing condition 1, 20 °C and 95% RH; (**d**–**f**) 28 curing days at curing condition 2, 60 °C and 100% RH.

Mortars prepared with HAC showed a porosity lower than that of the PC mortars (13.1% vs. 14.9% of total porosity, respectively), but with a different pore size distribution. In HAC mortars, hardened under curing condition 1 (20 °C and 95% RH), the pattern obtained by MIP was bimodal (Figure 9), with two main pore populations around 0.15 and 2.5 μm, both related to large capillaries.

**Figure 10:**
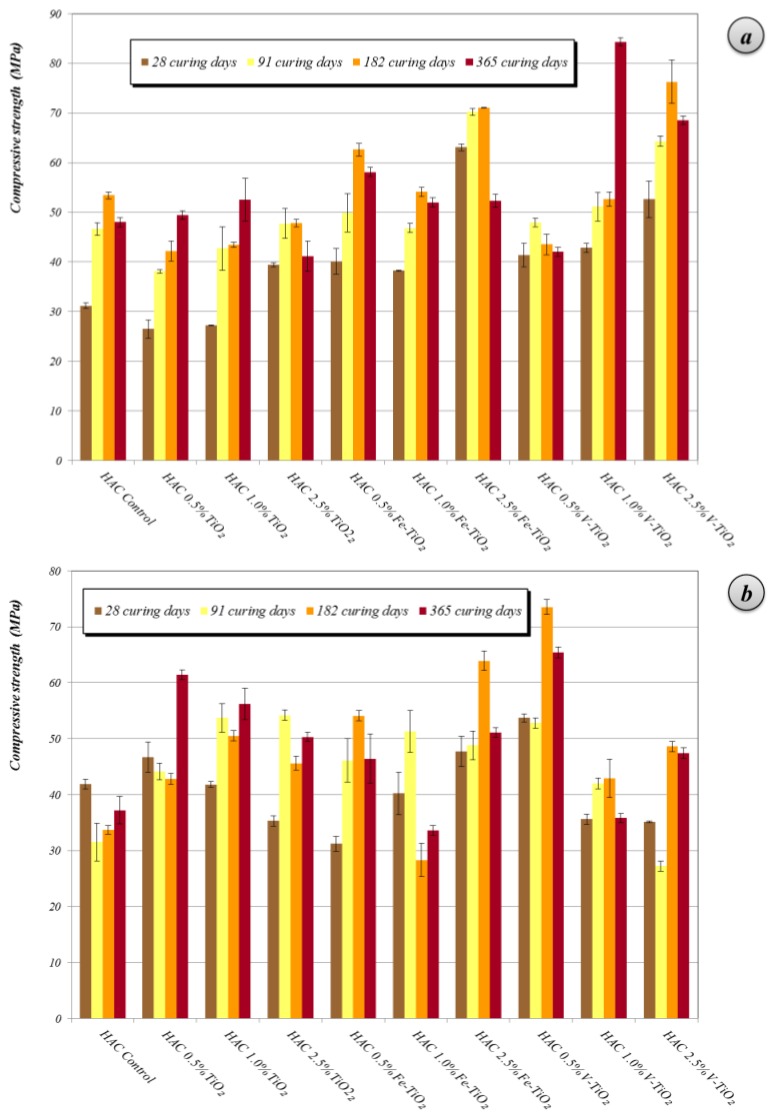
Compressive strengths at different curing times of HAC mortars: (**a**) Curing 20 °C, 95% RH; (**b**) Curing 60 °C, 100% RH, with nano-structured photocatalysts.

These mortars showed a small amount of medium and gel capillaries. Furthermore, they also presented significant porosity above 10 μm, usually ascribed to entrained air. The incorporation of bare titania reduced the amount of large capillaries, increasing—as a result of the filler effect—the medium capillaries. This reduction of the large capillaries was also observed for Fe-TiO_2_ and V-TiO_2_ samples, and accounted for the enhancement of the compressive strengths of these mortars (Figure 10a). In these mortars (curing condition 1), the effect of the different mixing water amounts was masked by the stronger influence of the filler effect of the additives: for example, V-TiO_2_ samples, with the largest mixing water amounts, showed higher compressive values than the control group. Even more, at longer curing times (one year), V-TiO_2_ mortars presented the highest compressive strength values of all assayed HAC mortars. On the other hand, Fe-TiO_2_, with the lowest mixing water amounts, yielded, after 28 curing days, similar compressive values to the V-TiO_2_ mortars. However, bare TiO_2_ mortars (0.5% and 1%), with the same mixing water amount as the control group, exhibited lower compressive strengths.

The second curing condition, 60 °C and 100% RH, favored the formation of stable calcium aluminates, denser than the metastable compounds, resulting in a porosity increase (from 13.1 % to 17.8% of the total porosity for plain HAC mortars) and subsequent lower compressive strengths (Figure 10b). This is the expected behavior of aged calcium aluminate cement due to the formation of the stable phases, which formation was induced forcedly by the curing condition 2. The most noticeable increments were seen in the range of large capillaries around 2 μm of diameter and in the range of the macro-pores above 10 μm (Figure 9). In these mortars with higher porosity, the incorporation of the photocatalytic additives yielded a clear filler effect, with a sharp reduction in the population of the large pore sizes (around and above 10 μm) and in the population of the large capillaries around 2.5 μm. A parallel increase in pores with a main peak between 0.2 and 0.35 μm was also observed. XRD confirmed the formation of stable calcium aluminate hydrates, as discussed above (see Table 4).

In these samples, the effect of the different mixing water amounts did not result in a clear relationship with the compressive strength performance: for example, mortars with 1% of V-TiO_2_, with the largest mixing water amount, yielded the largest compressive strength after 28 curing days, whereas the higher dosages of this additive (with the same mixing water percentage) resulted in lower compressive strengths than that of the control group.

##### SEM examination

**Figure 11:**
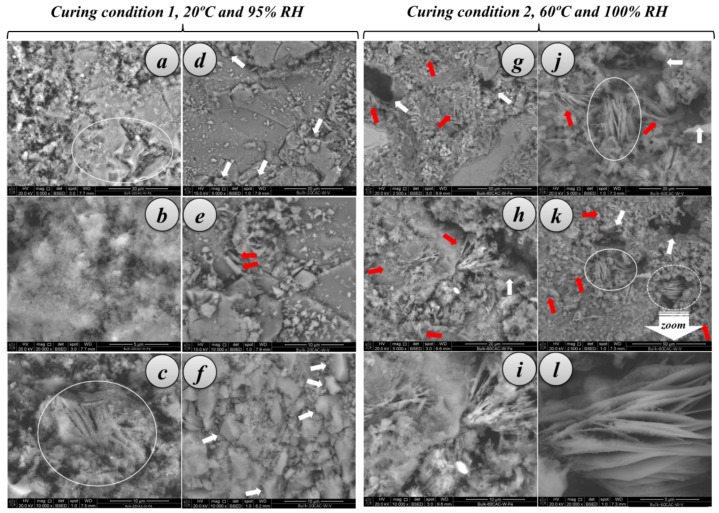
Microstructural examination of HAC mortars (28 curing days) with nano-structured additives (2.5 wt. %): (**a**–**c**) Micrographs for samples with Fe-TiO_2_ after curing condition 1 (20 °C and 95% RH); (**d**–**f**) Micrographs for samples with V-TiO_2_ after curing condition 1; (**g**–**i**) Micrographs for samples with Fe-TiO_2_ after curing condition 2 (60 °C and 100% RH); (**j**–**l**) Micrographs for samples with V-TiO_2_ after curing condition 2. An enlargement of the encircled dotted area in (**k**) is shown in (**l**).

Under curing condition 1, samples with Fe-TiO_2_ depicted an irregular pattern with a very low degree of crystallinity, in good agreement with the XRD (X-ray Diffraction) pattern results (Figure 11a). The microstructure of these mortars appears dominated by a gel-like structure (mainly composed of metastable hydrates and AH_3_, see Figure 11a) in which some trapezohedral or rhombo-trapezohedral crystals can be identified (outlined area in Figure 11a, ascribed to C_3_AH_6_ phases, although there was no evidences of cubic crystals) together with microcrystalline structures (spongy-shaped areas) and anhydrous phases (brighter areas in Figure 11b). Fibrous areas, which could be related to the growth of AH_3_ needles, were also seen in the samples with Fe-TiO_2_ (outlined area in Figure 11c).

Generally, these mortars showed low total porosity which is very much in line with the observations of the pore structure by MIP. Micrographs of mortars with V-TiO_2_ evidenced this low porosity (Figure 11d,e) and the combination of large amorphous crystals with clusters of trapezohedral crystals (denoted by arrows in Figure 11d,f). Some hexagonal prismatic crystals could also be identified as CAH_10_ metastable structures (red arrows in Figure 11e).

The second curing condition (higher temperature and relative humidity) depicts a different microstructural pattern of the mortars (Figure 11g–l). The presence of larger pores was remarkable, in accordance with the pore size distribution measurements, as a consequence of the formation of denser stable hydrates, which resulted in the formation of new large pores. Examples of these pores are indicated by white arrows in Figure 11g–k. This fact explains the fall in compressive strength of these mortars. The textural appearance was quite heterogeneous. The use of a Back Scattered Electron Detector (BSED) showed the presence of anhydrous compounds that remains un-hydrated (brighter spots in Figure 11i). In the microstructure the presence of needle-like crystals of stable AH_3_ was observed (red arrows, Figure 11g–k, and a detailed micrograph in Figure 11i). Furthermore, the SEM examination also showed, especially in the mortars with V-TiO_2_, some areas of stacked hexagonal thin layers that have been ascribed to C_2_AH_8_ and C_2_ASH_8_, stratinglite (thickness around 0.1 microns), according to the EDS elemental mapping analysis (Figure 11j,k, outlined areas with white ovals; Figure 11l shows a detailed examination of these piles of layered crystals). These compounds have been reported to be formed in CAC under hydration conditions similar to those of the curing condition 2 of the current work [38,39,40,41,42,43,44]. These phases grew at the expense of CAH_10_ and should be expected to convert into C_3_AH_6_ at later hydration stages. By XRD, V-TiO_2_ HAC mortars showed, under curing condition 2, a relatively low amount of C_3_AH_6_, in agreement with these findings. The absence of a clear formation of isomorphic cubic crystals of C_3_AH_6_ in these samples is also a further confirmation. To a certain extent, the addition of nano-structured additives, especially V-TiO_2_, hindered the conversion reaction, in line with the effect caused by other nano-materials that has been reported for CAC [45].

**Figure 12:**
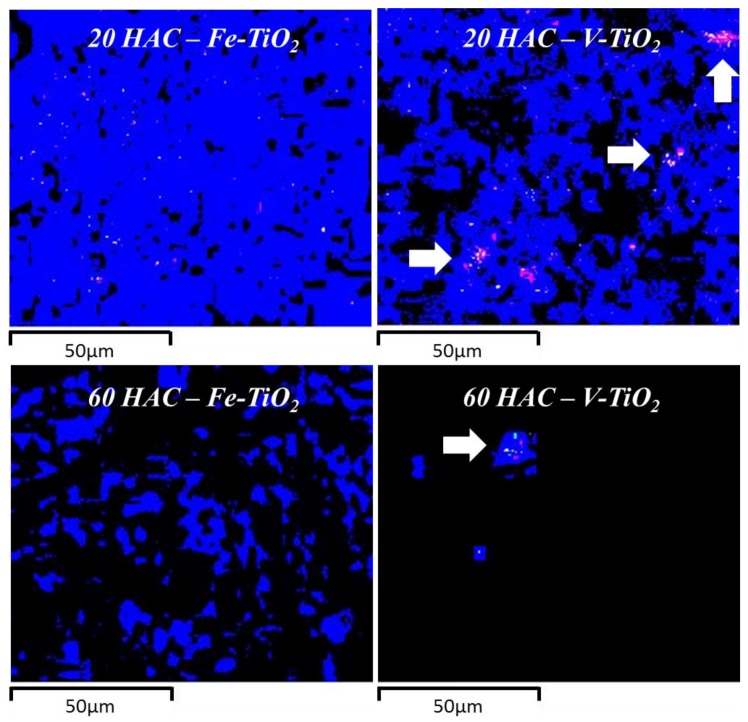
Comparative Ti distribution (SEM-EDS) of HAC mortars (after 28 curing days) with Fe-TiO_2_ and V-TiO_2_ nanostructured additives (included in 2.5 wt. %). For the purpose of clarity, due to the low ratio of Ti in the mortars, images were equally adjusted (brightness and contrast). Areas showing an accumulation of Ti in the V-TiO_2_ samples are indicated with arrows.

A comparative study of the distribution of the Fe-TiO_2_ and V-TiO_2_ nano-additives was carried out by EDS elemental mapping. It was seen that V-TiO_2_ in HAC mortars (Figure 12) was concentrated in specific areas in agreement with its tendency to agglomerate. Conversely, Fe-TiO_2_ showed a much more uniform distribution as can be seen in Figure 12.

### 2.4. Influence of the Nano-Photocatalysts on the Water Sorption Phenomena

When incorporated to cementing systems, besides the properties of the additives (particle size and hydrophilicity, as discussed above), the chemical and mineralogical composition of the binding matrix, its roughness as well as its pore size distribution, also have a strong influence on the wettability of the surface of the mortars. Quantitative water absorption was determined, which is important since in porous building materials an increased wettability could involve an increase in water absorption that would be a potential source of damage for these materials [46].

**Figure 13:**
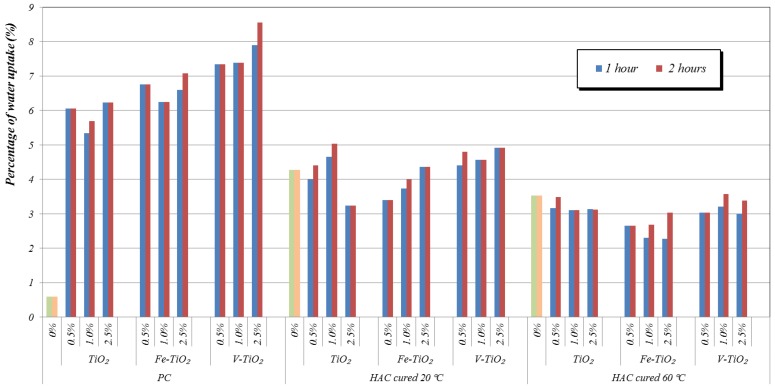
Water absorption of the mortars (after 28 curing days) in comparison with additive-free specimens (0%). Values measured after 1 and 2 h of water immersion.

Regarding the quantitative water absorption in the percentage depicted in Figure 13, the effect of the three different additives was similar, although doped additives showed slightly higher water absorptions than bare TiO_2_, in good agreement with their higher hydrophilicity determined by XPS. Nevertheless, the most significant changes should be ascribed to modifications in the pore structure. Similar values to those of the control samples were found for HAC mortars.

The highest increase in water absorption in comparison with the control sample was observed for PC samples. Changes can be ascribed to the modification of the pore structure. Particularly in these samples the increase in medium capillaries (pores between 0.05 and 0.01 μm) can account for these results, since these pores are responsible for the permeability of the mortars. Adjustment of the final pore structure of these PC mortars should then be carefully considered after the addition of nanostructured additives.

The main conclusion that can be drawn is that the presence of the photocatalysts incorporated as bulk addition in HAC mortars did not significantly increase either the water absorption rate or the percentage of absorbed water due to their higher hydrophilic character. This is largely beneficial for the practical application of these systems, under condition of pore structure refinement.

## 3. Materials and Methods

### 3.1. Materials

Two different binders and a siliceous sand as aggregate were selected to prepare the mortars. The mineralogical and granulometric composition of the sand has been reported elsewhere [47]. The different binding materials were:

(a) Portland Cement (PC) (type CEM II32.5 N, Portland, OR, USA),
(b) High alumina cement, (HAC) (Ternal White, Kerneos, Puteaux, France).

Three photocatalysts were used: bare TiO_2_ (Aeroxide P25, Evonik, Essen, Germany) and Fe-TiO_2_ and V-TiO_2_ (synthesized by flame spray pyrolysis, FSP [47], and supplied by Centro Tecnológico L’Urederra, Los Arcos, Spain). According to the data provided by the supplier, Fe-TiO_2_ was obtained by a solution of Fe^3+^ ion as precursor, whereas V-TiO_2_ was obtained from a V_2_O_5_-TiO_2_ mixture.

### 3.2. Methods

#### 3.2.1. Characterization of the Photocatalytic Additives

Specific surface area was determined in samples (0.2 g) by the BET method (ASAP 2020 equipment, Micromeritics, Norcross, GA, USA) studying N_2_ adsorption at 77 K, using a degassing temperature of 150 °C. XRD and XRF (X-ray Fluorescence) were respectively used to study the mineralogical and chemical compositions of the additives. The refinement of the XRD patterns was carried out by using the software of the equipment. The microstructure of the nano-structured additives was also examined in an energy filter transmission electron microscope (EFTEM, Libra 120 Zeiss GmbH, Oberkochen, Germany). Images were obtained by using the software iTEM 5.1 (Olympus Soft Imaging Solutions GmbH, Münster, Germany). Particle size observed by TEM examination agreed well with the results provided by XRD, although polydispersity of the particles made the particle size measurements by TEM less reliable than by XRD.

The surface chemical composition of the powders was investigated by X-ray photoelectron spectroscopy (XPS). For this purpose, ESACALAB 200-X with ECLIPSE data-system (VG Scientific, Waltham, MA, USA) and MgK-alpha excitation equipment was employed. Special attention was paid to the hydrophilicity of the nano-additives, assessed by the deconvolution of the O region.

Particle size distributions as well as the zeta potential measurements of the suspensions of the additives (1 wt. %) were determined using a Nanozeta Sizer (Malvern Instruments Ltd., Worcestershire, UK) in synthetic pore cement solution (pH 12.5 and 1% CaCl_2_).

#### 3.2.2. Mortar Preparation and Curing Regimes

Samples were prepared with a binder:aggregate ratio of 1:3 by weight (with variable binder:water ratio that was adjusted for each fresh mixture in order to ensure a good workability, as measured by a fixed spread of 175 ± 10 mm in the flow table test—see below for details about this test —). This procedure, related to the variability of the mixing water, is well established and has been thoroughly used in the field just to guarantee the workability and application of the mixtures. Otherwise, with a fixed amount of mixing water, some mixtures would simply be unworkable (either by poor consistency or by disaggregation caused by a lack of enough mixing water). Three different amounts of each photocatalyst (TiO_2_, Fe-TiO_2_ and V-TiO_2_) were studied: 0.5 wt. %, 1 wt. % and 2.5 wt. % with respect to the binder weight.

The raw components, binder, aggregate and nano-additives, were blended for 10 min at low speed in a Proeti ETI 26.0072 mixer (Madrid, Spain). Water was then added and mixed for 90 s. Then, the properties of the fresh state were determined as described below. Afterwards, mortars were cast in cylindrical moulds (36 mm height and 40 mm diameter) and de-moulded 24 h later. Curing conditions were as indicated:

- PC moulds were cured at 20 °C and 95% RH (Relative Humidity).
- Mortars of HAC were subjected to two distinct curing regimes: curing condition 1 was the same as that used for PC mortars, in which calcium aluminate cements are expected to develop metastable hydrates. The parameters for curing condition 2 were 60 °C and 100% RH: under such conditions, calcium aluminate cements yield cubic, stable calcium aluminate hydrates [48]. This curing condition is an accelerated way of obtaining stable phases. This knowledge is relevant to check the performance of these materials once they are aged involving the well-known conversion of metastable hydrated phases into the stable compounds.

Hardened samples were further characterized after different curing periods, as described below. Considering the relatively low percentages of the additives, the final color of the prepared specimens did not undergo any changes after the additive incorporation. In order to make the results representative, mortars were tested at each curing time in triplicate.

#### 3.2.3. Characterization of the Mortars with Photocatalysts

In the plastic state, consistency was measured by means of the flow table test [49]. A truncated metallic cone was filled with the sample; then, the metallic cone was removed and the cone-shaped sample was vertically lifted (15 strokes of the flow table) so that gravity allowed the sample to slump down and a quantitative measure of the spread was recorded.

Isothermal calorimetry studies (TAM Air equipment, TA Instruments, New Castle, DE, USA) were also conducted with the aim of studying the changes in the hydration process. Different amounts (<3 g) of the powder binding material were mixed with the required percentage of photocatalytic additives without implying any replacement of the mass of cement. Afterwards, samples were stirred with water at a suitable water/binder ratio (0.5 for PC and 0.37 for HAC water/cement ratios). Isothermal calorimetry data were collected for 24 h at 25 °C. A Vicat needle apparatus, according to a standardized procedure [50] was used to study the setting time.

In hardened samples, compressive strengths were measured at a loading rate of 50 N s^−1^ at different curing ages: 28, 91, 182 and 365 days. Pore size distributions were obtained by mercury intrusion porosimetry (MIP) (Micromeritics-AutoPoreIV-9500; pressure 0.0015 to 207 MPa). Complementary characterization by XRD (Bruker D8 CuKα1, Billerica, MA, USA, from 2° to 80° 2θ; increment of 0.02° at 1 step s^−1^ scan rate) was carried out by means of the software of the equipment. The ratio of the heights of the signals of the most intense diffraction peaks was used as a semi-quantitative indication of the relative amount of the different crystalline compounds. TG-DTA (TG-sDTA Mettler Toledo 851^e^, Columbus, OH, USA, alumina pans, from 25° to 1100 °C, under static air atmosphere with a purge of 20 mL min^−1^ of N_2_) analyses were also done to fully characterize the mixtures.

Scanning Electron Microscopy combined with energy-dispersive X-ray microanalysis (SEM-EDS, FEI Instrument, Quanta 3D FEG, with INCA IE 350 Penta FET X-3 EDS, Oxford Instruments, Abingdon, UK) was used for microstructural examination and elemental mapping of the mortars modified with the nano-structured additives.

Water absorption capability of the mortars was determined by immersing the samples into distilled water. Sample weights were measured at hour 0 (before immersing), and 1 and 2 h. Water absorption capability was calculated by the difference of weight in percentage. In all instances, three replicates of the different tests were carried out and the reported results are the average values.

## 4. Conclusions

Different mortars with nano-structured photocatalysts in bulk were prepared and their plastic and hardened performances were studied. The incorporation of the nano-additives showed a tendency to increase the water demand of the mortars, which was especially true for the addition of V-TiO_2_. This fact, which was further confirmed by particle size measurements, was due to its high specific surface area and its tendency to agglomerate, thus reducing the fluidity of the plastic paste.

All the nano-structured additives accelerated the hydration process of the cementitious binders due to the presence of additional nucleation sites, as proven by isothermal calorimetry studies. There was no evidence of pozzolanic reaction of any of the additives.

The incorporation of the nano-structured additives had a different influence on the compressive strengths. As compared with additive-free mortars, PC mortars displayed a decrease due to adverse changes in the pore size, interferences in the hydration of cementitious phases and the increase in the amount of mixing water. In PC mortars, Fe-TiO_2_ and V-TiO_2_, after a one year curing period, yielded the highest compressive strengths due to the filler effect and the subsequent porosity reduction. The microstructural examination of these mortars showed the formation of hollow shell grains responsible for the predominant capillary pore structure.

The nano-additives enhanced the compressive strengths of HAC mortars. The incorporation of photocatalytic additives reduced the large capillaries due to the filler effect, thus raising compressive strengths. Microstructural examination by SEM showed, after curing condition 1 at 20 °C and 95% RH, the predominance of gel-like structure in HAC mortars, combined with trapezohedral crystals and microcristaline areas. The curing condition 2 at 60 °C and 100% RH of these mortars was seen to favour the formation of denser stable calcium aluminate hydrates, increasing the porosity of the samples with a parallel reduction in strength. This fact was confirmed by SEM examination. Needle-like structures of AH_3_ were found in HAC mortars. V-TiO_2_ was found to hinder the formation of stable hydrates, and the conversion reaction was hindered showing C_2_AH_8_/C_2_ASH_8_ hexagonal layers. Elemental mapping showed a strong tendency of V-TiO_2_ to agglomerate.

The percentages of water uptake of the treated mortars showed that similar values to those of the control mortars were found for HAC mortars. For PC mortars the absorption of water increased as a consequence of the rise of medium capillaries, so that a refinement of the pore structure would be necessary to avoid a large wettability.

According to the results of hydration times, compressive strengths and microstructural characteristics, HAC was proven to be the best assayed binder to immobilize the nano-additives for photocatalytic purposes.

## Figures and Tables

**Table 1:** Chemical and mineralogical compositions of the raw cementing phases.

Binding Phase	Mineralogical Phases		Chemical Composition					
	Main	Minor	Al_2_O_3_ (%)	CaO (%)	Fe_2_O_3_ (%)	SiO_2_ (%)	SO_3_ (%)	Na_2_O + K_2_O (%)
PC	Ca_3_SiO_5_ (C_3_S), Ca_2_SiO_4_ (C_2_S)	Ca_3_Al_2_O_6_ (C_3_A), Ca_4_Al_2_Fe_2_O_10_ (C_4_AF), CaSO_4_·0.5H_2_O	4.0	62.0	4.0	20.0	1.6	0.3
HAC	CaAl_2_O_4_ (CA), CaAl_4_O_8_ (CA_2_)	Ca_12_Al_14_O_33_ (C_12_A_7_), CaCO_3_ (C)	70.5	28.5	0.2	0.6	<0.3	<0.5

**Table 2:** Characteristics of the photocatalytic additives.

Photocatalytic Compound	Anatase (%)	Rutile (%)	Density (g cm^−3^)	Specific Surface Area (m^2^ g^−1^)	Particle Size (nm)
TiO_2_ *	78.8	21.2	4.3	50	21
Fe-TiO_2_	69.1	30.9	3.9	101	16
V-TiO_2_	77.5	22.5	3.4	113	15

* Density value provided by the manufacturer.

**Table 3:** Water demand of different samples expressed as water/cement ratios.

Sample	Control	TiO_2_			Fe-TiO_2_			V-TiO_2_		
	0%	0.5%	1.0%	2.5%	0.5%	1.0%	2.5%	0.5%	1.0%	2.5%
PC	0.35	0.35	0.38	0.38	0.36	0.36	0.37	0.41	0.41	0.41
HAC	0.35	0.35	0.35	0.36	0.33	0.33	0.33	0.36	0.36	0.36

**Table 4:** Semi-quantitative XRD phase assemblage of aluminate phases and nano-additives in HAC samples after 28 days of curing condition 1 (20 °C and 95% Relative Humidity (RH)) and curing condition 2 (60 °C and 100% RH).

**Sample**	**Curing Condition 1**							
	**CA**	**C_12_A_7_**	**C_3_A**	**CAH_10_**	**C_3_AH_6_**	**AH_3_**	**Anatase**	**Rutile**
HAC Control	*	*	-	-	-	-	-	-
HAC TiO_2_	s	s	-	-	-	-	s	-
HAC Fe-TiO_2_	*	s	-	-	-	-	s	-
HAC V-TiO_2_	s	s	-	-	-	-	s	-
**Sample**	**Curing Condition 2**							
	**CA**	**C_12_A_7_**	**C_3_A**	**CAH_10_**	**C_3_AH_6_**	**AH_3_**	**Anatase**	**Rutile**
HAC Control	-	-	-	-	s	s	-	-
HAC TiO_2_	-	-	-	-	s	s	-	-
HAC Fe-TiO_2_	-	-	-	-	s	s	s	s
HAC V-TiO_2_	-	-	-	-	s	*	-	-

-: Non detectable; s: Small amount (less than 5 wt. %); * Significant amount (5–15%).
